# mTORC2 Is Activated under Hypoxia and Could Support Chronic Myeloid Leukemia Stem Cells

**DOI:** 10.3390/ijms24021234

**Published:** 2023-01-08

**Authors:** Cristina Panuzzo, Lucrezia Pironi, Alessandro Maglione, Simone Rocco, Serena Stanga, Chiara Riganti, Joanna Kopecka, Muhammad Shahzad Ali, Barbara Pergolizzi, Enrico Bracco, Daniela Cilloni

**Affiliations:** 1 Department of Clinical and Biological Sciences, University of Turin, 10043 Turin, Italy; 2 Department of Neuroscience Rita Levi Montalcini, Neuroscience Institute Cavalieri Ottolenghi, University of Turin, 10043 Turin, Italy; 3 Department of Oncology, University of Turin, 10043 Turin, Italy

**Keywords:** hypoxia, mTORC2 complex, phospho-Akt (Ser473), leukemic stem cells, Rictor, TKI, resistance

## Abstract

Hypoxia is a critical condition that governs survival, self-renewal, quiescence, metabolic shift and refractoriness to leukemic stem cell (LSC) therapy. The present study aims to investigate the hypoxia-driven regulation of the mammalian Target of the Rapamycin-2 (mTORC2) complex to unravel it as a novel potential target in chronic myeloid leukemia (CML) therapeutic strategies. After inducing hypoxia in a CML cell line model, we investigated the activities of mTORC1 and mTORC2. Surprisingly, we detected a significant activation of mTORC2 at the expense of mTORC1, accompanied by the nuclear localization of the main substrate phospho-Akt (Ser473). Moreover, the Gene Ontology analysis of CML patients’ CD34+ cells showed enrichment in the mTORC2 signature, further strengthening our data. The deregulation of mTOR complexes highlights how hypoxia could be crucial in CML development. In conclusion, we propose a mechanism by which CML cells residing under a low-oxygen tension, i.e., in the leukemia quiescent LSCs, singularly regulate the mTORC2 and its downstream effectors.

## 1. Introduction

Chronic myeloid leukemia (CML) is a clonal myeloproliferative disorder characterized by a peculiar cytogenetic abnormality, known as the Philadelphia chromosome (Ph) [1,2]. As a result, part of the breakpoint cluster region (*BCR*) gene fuses with Abelson kinase (*c-ABL*), giving rise to the oncogene BCR-ABL. The constitutive active tyrosine kinase BCR-ABL triggers a cascade of signals that control the cell cycle, proliferation, and DNA damage repair. Over the last couple of decades, tyrosine kinase inhibitors (TKIs) selectively targeting BCR-ABL have revolutionized the treatment of several cancers, including CML. Indeed, since the arrival of TKIs, the remission rate among CML patients has increased dramatically. Nevertheless, relapse can still occur because the leukemic stem/progenitor cells (LS/PCs) responsible for the onset of the disease and reside in the bone marrow niche are insensitive to TKIs [3,4,5]. LS/PCs are characterized by their almost unlimited self-renewal and low proliferation rate and thus play a pivotal role in sustaining the bulk of leukemic cells. Therefore, a current major challenge is the identification and characterization of the signaling pathways regulating LS/PCs activity to identify novel therapeutic approaches to specifically target and eradicate them. In recent years, a detailed characterization of hematopoietic stem cells (HSCs) has been carried out, highlighting the important role of the hypoxic niche to support the quiescent stem cell functions such as cell cycle control, survival, metabolism, and protection against oxidative stress. As CML is a clonal disorder, it is not surprising that the acquired TKIs resistance originates from LSCs [6,7]. Consistently, inquiries aimed at ascertaining the molecular processes contributing to the sustainability of LSCs unveiled that the mammalian Target of Rapamycin (mTOR) signaling pathway contributed significantly [8,9]. mTOR is a serine/threonine (Ser/Thr) kinase that exists in two distinct multisubunit complexes with nonoverlapping functions: mTOR complex 1 (mTORC1) and mTOR complex 2 (mTORC2). Both complexes act by integrating extracellular with intracellular signals, controlling a variety of cellular functions (e.g., growth, proliferation, differentiation and survival), and culminate with the phosphorylation of some members of the Ser/Thr protein kinase A, G and C (AGC) subfamily, including Akt and the ribosomal protein S6 kinase (S6K) [10,11,12]. Therapeutically, rapamycin was formerly considered the gold-standard inhibitor for mTOR but later has been seen as rather unsatisfactory because of its ability to silence mTORC1 although TORC2 remains still active, thus enabling the triggering of survival kinase Akt [13]. Accordingly, alternative therapeutic approaches have been proposed to overcome rapamycin’s limits.

The association of rapamycin with the adenosine monophosphate (AMP)-mimetic compound (5-aminoimidazole-4-carboxamide-1-b-4-ribofuranoside(AICAR)) leads to the suppression of Phospho-Lipase D (PLD) activity, which in turns lowers the phosphatidic acid (PA) levels and destabilize the mTORC2 complex by making it sensitive to tolerated doses of rapamycin [14,15].

In this regard, Akt, a central mediator of the Phosphoinositide 3-kinases (PI3K) pathway and an mTORC2 substrate, is crucial in controlling several cellular functions. It is well recognized that the AKT phosphorylation status is controlled by balancing the activities of kinases (i.e., 3-Phosphoinositide-dependent kinase 1 (PDK-1) and mTORC2) and phosphatases (i.e., Protein phosphatase 2 (PP2A) and PH domain and Leucine rich repeat Protein Phosphatases (PHLPP)). Although the functions of these enzymes are well known and evolutionary conserved, much remains to be understood, as suggested by the insights from lower eukaryotes and genetically amenable model organisms. For example, in *Dictyostelium,* a mutant defective in one mTORC2 component, *PIANISSIMO*, the PKB/AKT phosphorylation is completely rescued after the silencing of a member of the conserved Hect-E3 ubiquitin ligase [16,17], thus suggesting that Akt regulation might be under the control of potential novel regulators other than the classical ones, such as the ubiquitin–proteasome system (UPS). Once PKB/AKT is activated, it may translocate to various subcellular compartments and further mediate several enzymatic biological effects [18]. The dysregulation of mTORCs signaling is a common feature of several cancers, including CML [19,20].

Indeed, the constitutive tyrosine kinase activity of BCR-ABL leads to the direct activation of mTORC1. Consistently, the inhibition of both mTORCs synergizes with imatinib to induce apoptosis, thus overcoming TKIs resistance [21,22,23]. In contrast, under physiological conditions, mTORC2 is essential for the stem cell maintenance and development/self-renewal but not for HSC differentiation [24,25]. Despite these findings, the role of mTORC2 in Ph+ leukemic stem cells has been poorly explored thus far.

In light of the above evidence, we decided to explore the role of mTORC2 in Ph+ cells under hypoxic condition, because low oxygen tension is a shared niche characteristic crucial for maintaining quiescence in many cancer stem cells, including LC/PCs. In the present study, for the first time we report the major role played by mTORC2-Akt signaling, at the expense of mTORC1, in the Ph+ cell line under low oxygen tension. The outcome of the inquiry unveiled (i) a sharp activation of the mTORC2 complex under hypoxic conditions accompanied by a reduction in cell growth and proliferation rate and by a switch toward a metabolic dormant state, and (ii) the subcellular relocalization of Akt within the nuclear compartment.

## 2. Results

### 2.1. Hypoxia Microenvironment Activates a Quiescent State in Ph+ Cell Line

**Figure 1:**
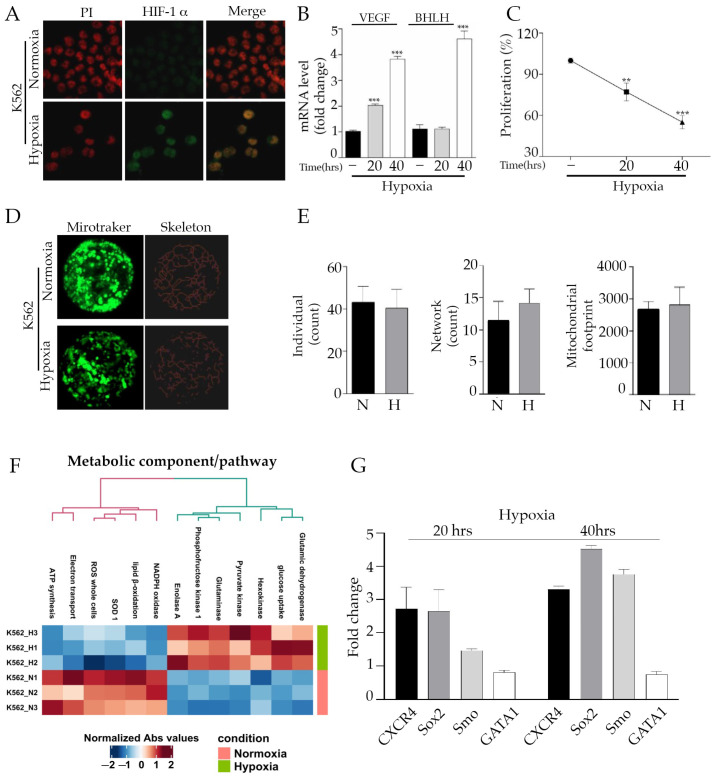
Hypoxia activates a quiescent state in Philadelphia-positive (Ph+) cell line: (**A**) Immunofluorescence of K562 cells incubated under hypoxic conditions for 20 and 40 h. The green nuclear staining corresponding to HIF-1α, a known marker of hypoxia, was registered after 40 h of low oxygen. Red propidium was used for nuclei staining. (**B**) Vascular endothelial growth factor (*VEGF*) and HIF-1 α Subunit (*BHLH*) gene expression was assayed by quantitative reverse transcription PCR (qRT-PCR) in K562 cells under normoxic and hypoxic conditions. The significant rise observed was indicative of low oxygen tension. (**C**) K562 cells under hypoxic conditions for 20 and 40 h were subjected to MTT assay to evaluate the proliferation index. The percentage of proliferation is expressed after normalizing with normoxia cells (100%). (**D**) The original green image obtained with confocal microscope is a Z-stack reconstruction image created (63× magnification) after mitotraker staining. It has been processed by MiNA toolset to draw an accurate skeleton of the mitochondria within the cell. (**E**) The number of individuals (unbranched structures), the number of networks (mitochondrial branched structures) and the mitochondrial footprint (the area occupied by mitochondrial structures) are unchanged between hypoxia vs. normoxia conditions. (**F**) Metabolic analysis profile of normoxic and hypoxic conditions. The results of a hierarchical clustering calculation applied on data are displayed in the heat map as a dendrogram. Heatmap represents processes with a significantly reduced activity in blue while processes with a significantly increased activity are in red. K562 H1, 2 and 3 corresponded to hypoxic condition triplicates. K562 N1, 2 and 3 represented the normoxic condition triplicates. (**G**) mRNA analysis of well-known stem cell markers (*CXCR4*, *SOX2*, *SMO* and *GATA-1*) in K562 cells under normoxia or hypoxia. Gene expression was represented as fold changes compared to normoxic conditions. ** *p* ≤ 0.01 and *** *p* ≤ 0.001.

To mimic the hypoxic bone marrow (BM) microenvironment, we incubated K562 cells under hypoxic conditions (1% oxygen atmosphere) for 20 and 40 h (h). Firstly, we verified the protein expression of the hypoxia inducible factor-1 α (HIF-1 α) as a marker of hypoxic status [26]. As shown by the immunofluorescence experiment (Figure 1A), after 40 h under hypoxic conditions, a significant accumulation of HIF-1 α within the nuclear compartment was observed, as compared to the control.

Afterwards, we used quantitative reverse transcription PCR (RT-qPCR) to measure the gene expression of vascular endothelial growth factor (*VEGF*), a direct target of HIF-1 transcriptional activity, and the HIF-1 α Subunit (Basic Helix–Loop–Helix Transcription Factor: *BHLH*), observing a time-dependent increase in both transcripts under hypoxic conditions (Figure 1B) [27]. The hypoxia treatment impaired K562 cells proliferation in a time-dependent manner (Figure 1C) without affecting the healthy state of cells, as confirmed by the calcein-AM staining and by preserving an intact mitochondrial network in which the distribution and organization of mitochondria was not rearranged nor fragmented (Supplementary Figure S1A) [28]. Furthermore, we performed a detailed panel of metabolic analysis after the cytosolic/mitochondria separation. The results obtained suggested that hypoxia can activate the glycolytic machinery in K562 cells, as revealed by glucose uptake, hexokinase, phosphofructose kinase 1, enolase A and pyruvate kinase increased activity. In contrast, the electron transport chain, ATP synthesis, lipid β-oxidation and total and mitochondrial reactive oxygen species (ROS) production was heavily reduced (Figure 1E), suggesting that environmental conditions such as low oxygen deprivation shifted the metabolism toward a glycolic pathway, shutting down oxidative phosphorylation (OXPHOS). Remarkably, our findings are in line with the prototypical hallmark of cancer stem cells (CSCs), where glycolysis is sustained at the expense of OXPHOS to avoid ROS production, which is dangerous for survival and self-renewal [29]. To further corroborate these early observations, the mRNA levels of known LSCs markers such as *SOX2*, *CXCR4* and *SMO* were measured, and a significant increase was observed under the hypoxic condition (Figure 1F) [30], while *GATA-1* levels, crucial for hematopoietic stem cell differentiation, were reduced. In addition, Sox2 was assessed in terms of protein level, and the obtained results confirmed the previous mRNA outcomes (Supplementary Figure S1B). All together, these data indicate that induced hypoxia may promote a reversible quiescent state in the leukemia cell line by miming the low oxygen tension of the BM niche.

### 2.2. Hypoxia Triggers mTORC2 Activation, Singular Akt Phosphorylation and Nuclear Localization

**Figure 2:**
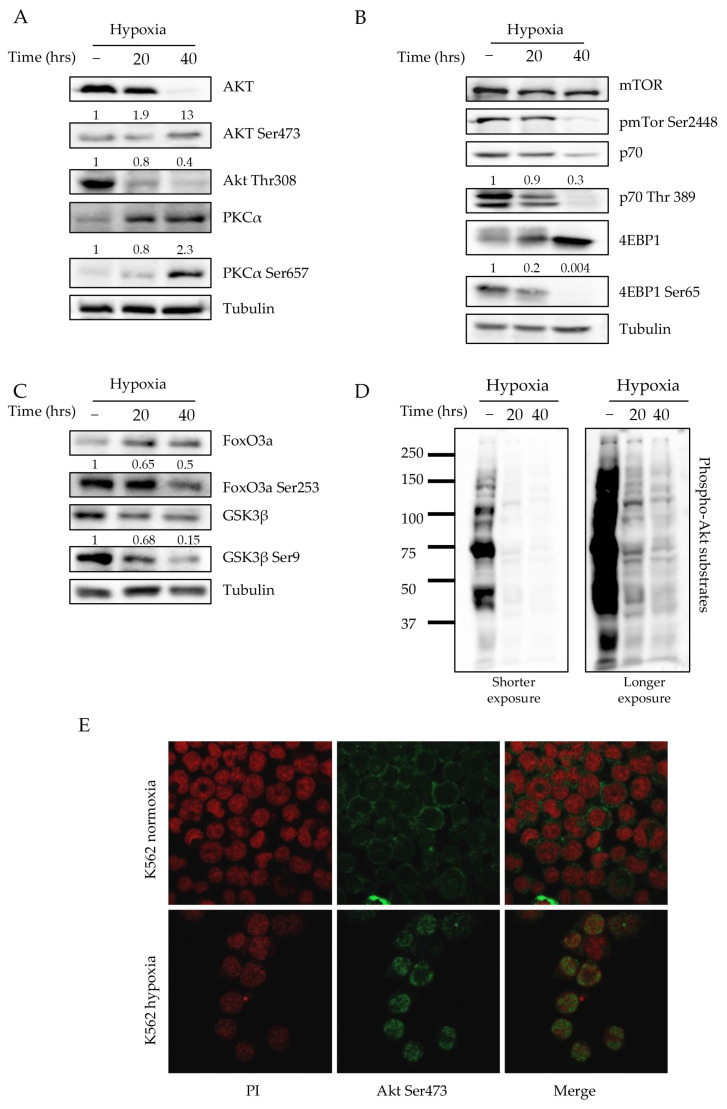
Hypoxia activates mTORC2 signaling and induces phospho-Akt (Ser473) nuclear localization: (**A**–**C**) Western blot analysis of the main mTORC1, mTORC2 and Akt kinase substrates. Numbers corresponding to normalization are expressed as fold changes compared to normoxic specimen. (**D**) Western blot analysis performed on K562 total cell lysates under normoxic and hypoxic condition by using a specific antibody to recognize the phospho-Akt-substrate consensus motif (R-X-R-X-X-S/T). Shorter and longer exposure were included to highlight the different patterns between our conditions. (**E**) Immunofluorescence of phospho-Akt (Ser473) on K562 cells incubated under hypoxic and normoxic conditions. Significant green nuclear staining corresponding to phospho-Akt (Ser473) was observed after low oxygen incubation. Red propidium iodide is used to label nuclei (63× magnification).

We next examined the effects of oxygen deprivation on mTORCs activity. Since mTORCs complexes display selectivity towards different substrates, Western blot analyses were used to assess the phosphorylation status of S6K, Akt and protein kinase C (PKC). Hypoxia induced a time-dependent increase in phospho-Akt (Ser473) and phospho-PKCα (Ser657) levels, which are well-known mTORC2 substrates (Figure 2A). On the contrary, phospho-p70 S6-K (Thr389) and phospho-4EBP1 (Ser65), the main mTORC1 substrates, appeared to be drastically reduced (Figure 2B). Interestingly, phospho-Akt (Thr308), a residue directly dependent on PDK-1, turned out to be unphosphorylated, thus suggesting the involvement of mTORC2 but not PDK-1 kinase [31]. To clarify this issue, we investigated the Akt substrates Forkhead box O3 (Foxo3a) and Glycogen Synthase Kinase 3 Beta (Gsk3β). Both are phosphorylated by Akt and thus their activity is downregulated.

The reduction in phospho-Foxo3a and phospho-Gsk3β observed after the hypoxia treatment suggests that low oxygen conditions activate these stem-related proteins and additionally confirm the quiescent status of K562 cells (Figure 2C) [32,33]. In fact, the inhibition of Gsk3β activity via Akt-mediated phosphorylation decreased glycogen synthesis and increased the accumulation of cyclin D1, facilitating the G1/S progression of the cell cycle. Meanwhile, Foxo3a inactivation via Akt phosphorylation on Ser253 residue reduced quiescence and decreased the repopulation potential of HSC. Our findings revealed that the Akt kinase activity towards the different substrates was widely modulable depending on the phosphorylation status of either Thr308 or Ser473 or both. Indeed, Foxo3a needs the full activation of Akt (both Thr308/Ser473 phosphorylated) to be phosphorylated, while the Gsk3β phosphorylation Akt-dependent mostly rely only on phospho-Akt (Thr308) residue.

By examining the levels of phosphorylation of Akt substrates in K562 cell lysates under hypoxic conditions by using a phosphor-specific antibody recognizing the degenerated Akt substrate peptide (R-X-R-X-X-S/T where X is any amino acid residue), we observed a sharp reduction in the phosphorylation pattern (Figure 2D) [34]. However, the presence of a few additional bands appearing under hypoxic conditions suggested the occurrence of substrates that were phospho-Akt (Ser473)-specific. Finally, we examined kinase localization under both normoxic and low-oxygen conditions. As shown in Figure 2E, hypoxia triggered the relocalization of Akt within the nuclear compartment. Interestingly, while such behavior was strictly dependent on Ser473 phosphorylation, it was fully independent from the phosphorylated Thr308 (Figure S1C).

### 2.3. Hypoxia Activates mTORC2 Signature in CD34+ of CML Patients

**Figure 3:**
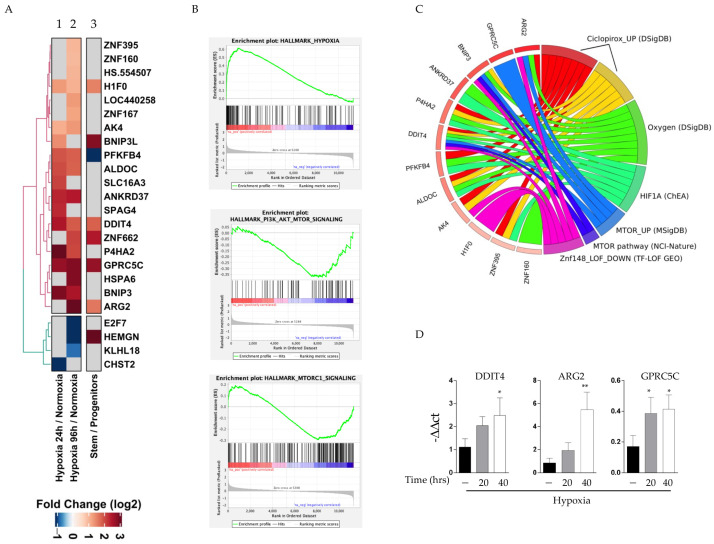
Hypoxia activates mTORC2 signature in CD34+ of CML: (**A**) Heatmap of hypoxic condition at 24 or 96 h with respect to their normoxic condition, showing the fold change, after log2 transformation, of differentially expressed genes (DEG) (column 1 and 2). Column 3: the overlap of DEGs induced by hypoxia with DEGs between hematopoietic stem and progenitor cells. (**B**) Enrichment plot hallmark of MSigDB. Profile of the Running ES Score and Positions of GeneSet Members on the log2FC ordered list of genes derived from differential expression analysis between CML CD34+ cells subjected to 96 h of hypoxia versus normoxia. (**C**) Circle plot of Gene Ontology analysis on DEGs (FDR adjusted *p*-value < 0.05). Circle plot shows the top significant terms obtained by GO analysis (on the right side of the circle). DEG relate to each GO term by a line when they occur in that term. Genes are ordered by log2FC. A box indicates the log2FC for each gene (on the left side of the circle). Only terms with at least three associated genes are shown. (**D**) Gene expression analysis of the more relevant DEGs (*DDIT4*, *ARG2* and *GPCR5C*) assayed by qRT-PCR in K562 cells under normoxic and hypoxic conditions. * *p* ≤ 0.5 and ** *p* ≤ 0.01.

In order to assess the relevance of mTORC2 signaling, we next attempted to analyze the gene expression profile of CD34+ cells derived from CML patients at diagnosis, 24 and 96 h after incubation under hypoxic conditions [35]. After the meta-analysis of the available data, we produced the derived heatmap showing the fold change, after a log2 transformation, of the differentially expressed genes (DEGs) in a low oxygen state at 24 and 96 h with respect to their normoxic conditions (Figure 3A, column 1 and 2). The log_2_Fold changes (FC)-ordered list of genes derived from the differential expression analysis between the CML CD34+ cells subjected to 96 h of hypoxia versus normoxia was used as input for Gene Set Enrichment Analysis (GSEA). Enrichment in the «hallmark gene sets» of the Molecular Signature Database (MSigDB) was computed using GSEA software. Seven gene sets were significantly enriched (*p* < 0.05) with a positive enrichment score (gene sets showing enrichment at the top of the ranked list), and 14 gene sets had a negative enrichment score (gene sets showing enrichment at the bottom of the ranked list) (Supplementary Figure S2A). Among the gene sets with positive enrichment, «HYPOXIA» was found (Figure 3B panel 1). Notably, among the gene sets with negative enrichment, «PI3K_AKT_MTOR_SIGNALING» and «MTORC1_SIGNALING» were found (Figure 3B panel 2 and 3). The fact that the mTORC1 pathway was one of the most significantly reduced pathways strengthens our in vitro results, suggesting a real implication of the mTOR pathway in the fate of stem cells. Moreover, it is not surprising that mTORC2 did not emerge, since this signaling is not found in the Molecular Signature Database.

To consolidate our evidence, a Gene Ontology analysis of DEGs resulted in a significant enrichment (False Discovery Rate—FDR—adjusted *p*-value < 0.05) of different terms related to metabolic alterations and adaptive responses to hypoxia (Figure 3B). Specifically, we found that enrichment in glycolysis (adj. *p*-value = 1.53 × 10^−2^) was represented by genes encoding for -Phosphofructo-2-Kinase/Fructose-2,6-Biphosphatase 4 (*PFKFB4*) and Aldolase Fructose-Bisphosphate C (*ALDOC*). *PFKFB4* and *ALDOC* genes together with Ankyrin Repeat Domain 37 (*ANKRD37*), Zinc Finger Protein 395 and 160 (*ZNF395* and *ZNF160*), Prolyl 4-Hydroxylase Subunit Alpha 2 (*P4HA2*) and the DNA Damage Inducible Transcript 4 (*DDIT4*) were predicted targets of the transcriptional regulator of the adaptive response to hypoxia (HIF-1 α). Interestingly, the mTOR pathway (adj. *p*-value = 1.76 × 10^−3^) appeared among the most relevant pathways under hypoxia. In detail, MTOR_UP, as reported in the circle plot, corresponded to a specific signature obtained in cells after the inactivation of the mTORC1 pathway, a condition that mimics the mTORC1 status observed in our specimens. The genes related to this process that arose after the analysis include *DDIT4*, *ARG2*, *GPRC5C* and *BNIP3*.

DDIT4, alias REDD1, directly regulates cell growth, proliferation and survival via the inhibition of the activity of mTORC1 [36,37].

ARG2 catalyzes the hydrolysis of arginine, and it has been associated with Rictor and mTORC2 in the process of controlling autophagy [38,39].

*GPRC5C* is an orphan gene encoding for type 3 G-protein-coupled receptor family C group 5 member C. mTOR signaling is directly activated by plasma membrane receptors, including GPCR family members, to whom GPRC5C also belongs [40].

BNIP3 and BNIP3L, being BCL2 interacting proteins, regulate apoptosis processes. These proteins are involved in mitochondrial quality control in response to mitochondrial damage. In addition, Bnip3, a hypoxia-inducible protein, directly decreases Rheb GTP levels and inhibits the mTORC1 pathway [41]. We subsequently investigated whether such effects identified in our analysis under in vitro hypoxia could reflect the stem cells compartment in CML patient cells, where a hypoxic environment represents the physiological condition of leukemic stem cells.

To broaden and validate our experimental data, we mined a GEO dataset obtained from hematopoietic progenitor cells (CD34+/CD38+) and hematopoietic stem cells (CD34+/CD38-), both derived from CML patients at diagnosis [42]. After comparing these two cell populations, we computed differential expression levels and obtained a list of 3170 DEGs. The overlap of DEGs induced by hypoxia and DEGs between hematopoietic stem and progenitor cells resulted in a short list of genes including *ARG2*, *DDIT4* and *GPRC5C* (Figure 3A column 3). Surprisingly, we investigated the pattern of activation of the above genes in our specimens, and they significantly increased after 20 and 40 h of hypoxia (Figure 3C).

## 3. Discussion

Bone marrow (BM) is a hypoxic compartment, with pO2 concentrations ranging from 1% to 4%. These conditions are crucial for the maintenance and pluripotency regulation of HSCs, as well as for their leukemic counterparts, LSCs [43]. The HIF transcription factor is a master regulator governing the survival, quiescence and metabolic processes of both HSCs and LCSs [44,45]. HIF-1 α stability and activity are tightly regulated by oxygen concentration. Hypoxic conditions drive its nuclear translocation, thus selectively enabling the transcription of genes bearing in their promoter hypoxia-responsive elements (HREs) [46]. In LCSs, a low oxygen concentration may either promote or impair different peculiar signaling pathways, leading to enhanced self-renewal, quiescence and refractoriness in TKIs [44,47]. In this regard, several mechanisms that are Bcr/Abl independent and relevant to LSC maintenance in CML have emerged in the last few years as potential targets to develop new therapeutic strategies to overcome TKI resistance. In the present study, we observed that mTORC2 appears to be active and phosphorylates Akt kinase under hypoxic conditions. Overall, our observations are consistent with previously published studies [48,49]. One interesting study suggests that mTORC2 may mediate several processes associated with migration, invasion and survival through PLD activation due to stress-dependent serum withdrawal. Remarkably, the latter activates HIF-1 α whereas mTORC2 is activated at the expense of mTORC1 [15,50].

Our findings lead us to speculate that in Ph+ cells under hypoxic conditions, the mTORC2-mediated Akt-dependent signaling might contribute to sustain the stemness features; thus, it represents as a potential target option for eradicating CML LSCs. After incubating K562 cells under hypoxia, we detected the activation of quiescent markers including the nuclear translocation of HIF-1 α, a reduction in the proliferation rate and an increase in *SOX2*, *CXCR4* and *SMO* transcripts amount. Furthermore, hypoxia-induced metabolic profiling exhibited a lowering in the OXPHOS activity that is a key stemness feature [29,51]. The in silico survey carried out on CD34+ CML cells after a meta-analysis of the available data revealed that a few genes are regulated under hypoxic conditions and some of them are associated with the mTOR pathways (e.g., *DDIT4*, *ARG2* and *GPRC5C*). Interestingly, DDIT4, also known as REDD1, is a direct transcriptional target of HIF-1α. DDIT4 positively regulates the activity of the Tuberous Sclerosis (TSC) complex (TSC1, TSC2 and TBC1D7), which in turn acts as a crucial negative regulator for the mTORC1 activity. Consistently, our findings unveil that the activity of mTORC1 is profoundly inhibited in K562 cells under hypoxic conditions. Furthermore, under stress conditions including hypoxia, amino acid starvation and DNA damage, DDIT4 promotes the activity of PP2A, which is responsible for the selective dephosphorylation of phospho-AktThr308 residue [52]. mTORC2 kinase appears to be enhanced by the withdrawal of amino acids (e.g., glutamine) and glucose, and it is essential in maintaining their correct flux through glucose- and glutamine-requiring biosynthetic pathways [53,54]. Hence, it is plausible to assume that the stem phenotype observed in the K562 CML cell line partly relies on the switching of the mTOR complexes activity. In favor of this hypothesis, different experimental evidence has revealed a central role of the Akt–mTOR network in HSC homeostasis [54]. The inactivation of mTORC2, through the silencing of *Rictor*, induces a proliferative boost in LSCs, indicating the vital role of mTORC2 in sustaining the hematopoietic stem cell compartment [24]. Accordingly, our findings display enhanced mTORC2 activity, as confirmed by the phosphorylation of two substrates, namely PKC alpha and Akt. Conversely, the mTORC1 substrates, S6K (pp70) and 4EBP1, are both heavily inhibited. Moreover, the fact that the main Akt substrates, Foxo3a and GSK3β, do not seem to be significantly phosphorylated under hypoxia, alongside with the pattern of the phospho-Akt-substrates, suggest that the Akt substrates’ selectivity and specificity is tightly associated with the kinase phosphorylation status. In K562 cells, PDK-1-dependent Akt phosphorylation (Thr308) appears to be inactive under hypoxia, in contrast to the mTORC2-dependent (Ser473) activation. To some extent, even though this feature has been described in other cellular contexts [55,56,57,58], our findings differ from those previously reported with murine models, in which PDK-1 appears to act as a pivotal player in regulating the function of HSCs [59]. It is highly likely that the conflicting results might be related to different kinds of HSCs (HSCs vs. LSCs) and experimental settings (in vivo vs. in vitro). Indeed, our experimental setting is over simplified compared with murine bone marrow, and previous inquiries refer to healthy HSCs. Nevertheless, currently it cannot be ruled out that the nuclear phospho-Akt (Ser473) is unable to phosphorylate substrates.

It has been shown that nuclear AKT supports the phenotype associated with the maintenance of CSCs, the regulation apoptosis, cell cycle progression, cell differentiation and DNA repair [60,61,62]. An open question that currently remains unanswered is whether the kinase moves into the nucleus in an already phosphorylated form. Remarkably, in aggressive variants of papillary thyroid carcinomas, mTORC2 has been demonstrated to localize in the nucleus, resulting in concomitant phospo-Akt (Ser473) increases [62]. Similarly, in the fission yeast *Schizosaccharomyces pombe*, TORC2 and AKT can both be found in the nucleus where, through direct interaction with E2F1, transcriptional activity is favored in response to DNA damage [63]. E2F1 has been proposed as a candidate for the regulation of LSCs quiescence in CML due to its role in controlling the G1-S phase transition and because of its increased expression at the level of CD34+ cells [64,65]. Finally, the regulatory effect on Akt and PP2A activity exerted by E2F1 has been reported [66]. Our bioinformatic survey revealed that among the downregulated genes, E2F7 acted as a direct suppressor of the transcriptional activity of E2F1, strengthening the hypothesis that an mTORC2-Akt-E2F1 axis might be involved in controlling CML-LSCs properties. Additionally, the overlap of DEGs induced by hypoxia in CML patients’ cells and DEGs between hematopoietic stem and progenitor cells resulted in a set of genes directly downstream of the mTORC2 pathway. Among the genes identified, the most attractive was GPRC5C, an orphan member of the type 3 G protein-coupled receptor family [40]. The role of GPCRs in embryonic development and stem cell maintenance has been recognized in the last decade. Increasing evidence has shown that CXCR4 is crucial for blood stem cell homing, as well as retention in the bone marrow. However, CXCR4 also controls the proliferation of nonhematopoietic stem cells. Although, the role of GPRC5 has recently been associated with dormant HSCs, where it acts by mediating ATRA action by restricting the protein translational rate and ROS levels [67]; to our knowledge, this is the first report where changes in *GPRC5C* gene expression have been associated to CML. Interestingly, *GPRC5C* expression has also been associated with the maintenance of the neuroblastoma cancer stem cell population, being a distinctive marker of the highly tumorigenic I-type stem cells. A previous study reported that GPCRs can inhibit mTORC1 activity in multiple cell lines and in mice via cAMP-PKA signaling, culminating in the phosphorylation of Raptor at Ser791, phospho-Akt (Thr308) inactivation and the subsequent deactivation of mTORC1 [68]. In this setting, phospho-Akt (Ser473) remains phosphorylated, supporting our data. Based on our experimental evidence, we propose a model in which mTORC2 and its main substrates are peculiarly activated in CML cells residing in a low oxygen environment, i.e., in leukemia quiescent stem cells, contributing to their resistance to current TKI-based therapies.

In this regard, mTORC1/mTORC2 inhibitors, belonging to the class of ATP-competitive inhibitors, are used in phase I and II studies. However, currently, selective mTORC2 inhibitors are not available. Consequently, the lack of tailor-made mTORC2 therapies means that it is difficult to evaluate their potential.

Based on our results and evidence from several other studies, we feel that identifying novel LSC strategies, novel substrates and novel pathways crucial for mTORC2 activity represents an attractive future challenge.

## 4. Materials and Methods

### 4.1. Cells Culture and Hypoxic Condition

A human K562 Ph+ cell line was purchased from the American Type Culture Collection (ATCC, Manassas, VA, USA) and was cultured in RPMI-1640 supplemented with 200 nmol/L Glutamine (EuroClone, Milan, Italy), 10% inactivated fetal bovine serum (FBS) (Sigma-Aldrich, St. Louis, MO, USA) and 0.1% penicillin/streptomycin (EuroClone). Cells were cultured at 37 °C in a humidified atmosphere flushed with 5% CO_2_ and, when needed, in a hypoxic (37 °C, 1% O_2_ and 5% CO_2_) incubator (InVIVO2 200 equipped with a Ruskinn Gas Mixer Q) for 20 or 40 h.

### 4.2. RNA Extraction and Quantitative Real-Time PCR (qRT-PCR)

Total RNA was extracted using TRIzol Reagent (Thermo Fisher Scientific, Waltham, MA, USA) according to the manufacturer’s instructions, and 1 μg of total RNA was reverse transcribed. *VEGF*, *BHLH*, *GPCR*, *DDIT4* and *ARG2* mRNA expression were evaluated with the SYBR Green approach (Thermo Fisher Scientific). Cycling conditions were as follows: 95 °C for 2 min, followed by 39 cycles at 95 °C for 5 s and 60 °C for 30 s. *SOX2*, *CXCR4*, *SMO* and *GATA-1* mRNA expression was evaluated using commercially available TaqMan probes assays (Thermo Fisher Scientific) according to the manufacturer’s instructions. Cycling conditions were performed in triplicate with the C1000 Thermal Cycler CFX96 Real-Time System (Bio-Rad, Hercules, CA, USA). Cts values were analyzed with Bio-Rad CFX Manager 3.1 software (Bio-Rad, Hercules, CA, USA) and were expressed after normalization with the *ABL* housekeeping gene. Universal human references RNA (Stratagene, San Diego, CA, USA) was used to calibrate the assay.

### 4.3. Cells Lysis and Western Blot

K562 cells were lysed in RIPA buffer on ice for 20 min (10% glycerol; 1% Triton X-100; 20 mM Hepes pH 7.4; 5 mM EDTA pH 7.2; 150 mM NaCl) and were supplied with protease and phosphatase inhibitors (1 mM Na_3_VO_4_, 1 mM PMSF, 2 µg/mL leupeptin, 2 µg/mL aprotinin, 2 µg/mL pepstatin). After centrifugation at 14,000× *g* for 15 min, the protein concentration was determined by using Bradford reagent (Bio-Rad), and 50 µg of total proteins were resolved in 4–15% gradient SDS-PAGE gels, and were subsequently transferred on PVDF filters. After blocking with 5% BSA (Sigma-Aldrich, St. Louis, MO, USA) in TBS 1x plus 0.3% Tween-20 (Sigma-Aldrich) for 1 h at room temperature (RT), immunoblots were incubated overnight (ON) at 4 °C with specific primary antibodies (mTor, p-mTor, p70, p-p70, 4EBP1, p-4EBP1, AKT, p-AKT Thr308, p-AKT ser473, p-PCKα, FoxO3a, p-FoxO3a, GSK3β e p-GSK3β, Cell Signaling Technology, Danvers, MA, USA). Peroxidise-conjugated secondary antibodies (Santa Cruz Biotechnology, Dallas, TX, USA) were used at 1:7000 dilution for 1 hour at RT. Protein detection was performed with an enhanced chemiluminescent reagent (Clarity Western ECL Substrate, Bio-Rad). An images analysis was performed using the Image Lab program (Bio-Rad).

### 4.4. Immunofluorescence Assay and Mitochondrial Analysis

Cytospins were prepared using 50,000 cells of K562 under hypoxic and normoxic conditions. Cells were fixed with 4% paraformaldehyde (PFA) (Sigma-Aldrich), permeabilized with 0.5% Triton (Bio-Rad), blocked for 45 min (10% fetal bovine serum, 5% BSA, 1% fish gelatine) and incubated ON at 4 °C with antibodies. Proteins detection was obtained via subsequent incubation with Alexa Fluor 488 secondary antibodies (Invitrogen, Waltham, MA, USA). PI (Sigma-Aldrich) was used for nuclear staining. Mounted slices with Mowiol were analyzed with a confocal scanning microscope (LSM 800; Carl Zeiss MicroImaging Inc., Oberkochen, Germany). The morphological analysis of mitochondrial networks after MitoTracker Freen staining was performed in silico as previously reported [28,69]. Images were captured using a 63X objective. The fluorescent signal was measured with image processing and was analyzed in the Image J program.

### 4.5. Hypoxia vs. Normoxia Gene Expression Analysis

The probes’ hybridization normalized intensity values were retrieved from the GEO series GSE48294 [35]. This dataset consisted of CD34+ bone marrow aspirates from three patients affected by chronic myeloid leukemia (CML). Limma package version 3.36.1 was used to compute the differential expression [70]. The probes were annotated using the Ensembl BioMart tool and the GRCh38.p12 as a reference genome for assembly [71]. In cases of missing annotation, the original annotation of the GPL10558 platform was used. To remove redundancy from multiple probes annotated by the same gene, we kept the probe with the lower adjusted p-value. Protein annotation was performed by retrieving the protein ID and details from the UniProt Knowledgebase (UniprotKB) hub [72]. The analysis was performed using R version 3.3.3 [73].

### 4.6. Gene Ontology Analysis of Hypoxia versus Normoxia Differentially Expressed Genes

Gene Ontology (GO) enrichment analysis was performed on differentially expressed genes after 96 h of hypoxic conditions, using the GEO series GSE48294 [35] and the tool EnrichR. A circle plot was created using the GOchord function of the GOplot package, version 1.0.2 [74]. Significant results for the Biological Process, Cellular component, Jensen compartments, Kyoto Encyclopedia of Genes and Genomes (KEGG) pathways 2016 and Chromatin Immunoprecipitation (ChIP) Enrichment Analysis (ChEA) 2016 were considered (FDR adjusted *p*-value < 0.1). The top 20 terms with lower significant adjusted p-values and at least three associated genes in the list of differentially expressed genes were used to generate the circular plot. The analysis was performed using R version 3.3.3.

### 4.7. Gene Expression Analysis of Hematopoietic Stem/Progenitor Cells

Probe hybridization–normalized intensity values of the GEO series GSE43754 [42] were analyzed with GEO2R, an online tool for differential expression analysis based on the GEOquery and Limma R packages. Hematopoietic progenitor cells (CD34+/CD38+) and hematopoietic stem cells (CD34 +CD38−) were derived from CML patients subjected to differential expression analysis with GEO2R. Probe identifiers were annotated using Ensembl Biomart software and GRCh38.p12 as the reference human genome. To remove redundancy on multiple probes annotated by the same gene, we kept the probe with the lower adjusted *p*-value.

Subsequently, the log2FC-ordered list of probes derived from the differential expression analysis of CML CD34+ cells subjected to 96 h of hypoxia versus normoxia was used as the input for the preranked GSEA versus hallmark gene sets of the Molecular Signature Database (MSigDB) using GSEA software with default parameters. Probes were listed using Illumina probe identifiers and were collapsed to gene symbols within the GSEA software using Human_Illumina_HumanHT_12_v4_BeadChip (Platform GPL10558) as the reference probe symbol identifier.

### 4.8. Proliferation Assay

Cell growth was evaluated with the 3-(4,5-dimethylthiazol-2-yl-2,5 diphenyl tetrazolium bromide (MTT) assay (Sigma-Aldrich) according to the manufacturer’s instructions. Therein, 30,000/well cells were seeded in triplicate, alone or with different drug concentrations, for 48 h. Then, 10 µL of MTT reagent was added to each well, and within 24 h the plate was analyzed with a microtiter plate reader (480 nm). The absorbance intensity was directly proportional to the number of viable cells. The experiments were performed in triplicate.

### 4.9. Metabolic Assays

#### 4.9.1. Mitochondria Isolation

Cytosolic/mitochondria separation was performed via differential centrifugation as described in [75]. In each fraction, the protein content was assessed with the BCA Protein Kit (Sigma-Aldrich). Cytosolic extracts were used to measure the enzymatic activity of glycolytic enzymes (hexokinase HK; phosphofructokinase-1, PFK-1, enolase A, pyruvate kinase, PK), cytosolic (total) ROS, NADPH oxidase (NOX), aldose reductase and superoxide dismutase (SOD) 1. Moreover, 100 μL of mitochondria extracts were used to measure the electron transport chain, ATP and mitochondrial ROS. To confirm the presence of mitochondrial proteins in the extracts, 10 μg of each sonicated sample were subjected to SDS-PAGE and probed with an VDAC/porin antibody (Abcam, Cambridge, UK).

#### 4.9.2. Glucose Uptake and Glycolytic Enzymes

The uptake of glucose was measured via radiolabeling whole cells with 1 µCi-deoxy-D-[^3^H]-glucose (PerkinElmer, Waltham, MA, USA) [76]. The results are expressed as picomoles of 2-deoxy-D-[3H]-glucose/mg cell proteins. The HK activity was measured with the Hexokinase Colorimetric Assay Kit (Sigma-Aldrich). The activities of the phosphofructokinease-1 (PFK1) assay and enolase were measured spectrophotometrically as reported in [77]. The activity of PK was detected with the Enzymatic Assay of Pyruvate Kinase kit (Sigma-Aldrich). The results are expressed as nmoles NADH/min/mg cell proteins (HK) or nmoles NAD^+^/min/mg cell proteins (PFK1, enolase, PK).

#### 4.9.3. Fatty Acid β-Oxidation

The rate of fatty acid β-oxidation was measured via radiolabeling mitochondria extracted from cells with 2 μCi [1-^14^C] palmitic acid (3.3 mCi/mmol; PerkinElmer). The amount of ^14^C-acid soluble metabolites (ASM) was quantified by liquid scintillation [78]. The results are expressed as picomoles of ^14^C-ASM/h/mg cell proteins.

#### 4.9.4. Glutaminolysis

Glutamine catabolism was measured as reported in [79]. Cells were washed with PBS, centrifuged at 13,000× *g* for 5 min at 4 °C, resuspended in 250 μL of buffer A (150 mM KH_2_PO_4_, 63 mM Tris/HCl, 0.25 mM EDTA; pH 8.6) and sonicated. Then, 100 μL of whole cell lysates were incubated for 30 min at 37 °C with 20 mmol/L L-glutamine in 850 μL of buffer B (80 mM Tris/HCl, 20 m NAD+, 20 mM ADP, 3% *v*/*v* H_2_O_2_; pH 9.4). The absorbance of NADH was monitored at 340 nm using a Lambda 3 spectrophotometer (PerkinElmer). The kinetics were linear throughout the assay. The results are expressed as micromoles of NADH/min/mg cell proteins and are considered as an index of the activity of glutaminase plus L-glutamic dehydrogenase. In a second series of samples, 20 μL of the glutaminase inhibitor bis-2-(5–phenylacetamido-1,3,4-thiadiazol-2-yl) ethyl sulfide BTPES (30 μM, inhibiting glutaminase activity at 100%) were added after 15 min. The absorbance of NADH was monitored for 15 min. The results, considered as an index of the activity of L-glutamic dehydrogenase, are expressed as micromoles of NADH/min/mg cell proteins. Glutaminase activity was obtained by subtracting the rate of the second assay from the rate of the first one.

#### 4.9.5. Electron Transport Chain

To measure the electron flux from complex I to complex III, taken as the index of mitochondrial respiratory activity, 50 μg of nonsonicated mitochondrial samples were resuspended in 0.2 mL of buffer A (5 mL KH_2_PO_4_, 5 mM MgCl_2_, 5% *w*/*v* bovine serum albumin), and 0.1 mL of buffer B (25% *w*/*v* saponin, 50 mM KH_2_PO_4_, 5 mM MgCl_2_, 5% *w*/*v* bovine serum albumin, 0.12 mM cytochrome c-oxidized form, 0.2 mM NaN_3_) was added for 5 min at room temperature. The reaction was started with 0.15 mmol/L NADH and was followed for 5 min, reading the absorbance at 550 nm with a Packard microplate reader EL340 (Bio-Tek Instruments, Winooski, VT, USA). The results are expressed as nanomoles of cytochrome c reduced/min/mg mitochondrial protein [75].

#### 4.9.6. ROS Measurement

The ROS amount in cytosolic extracts was measured by labeling samples with the ROS-sensitive fluorescent probe 5-(and-6)-chloromethyl-2′,7′-dichlorodihydro-fluorescein diacetate-acetoxymethyl ester (DCFDA-AM) [80]. The results are expressed as nmol/mg cell proteins.

#### 4.9.7. SOD Activity

The activity of cytosolic SOD1 was measured using 0.01 mg of proteins incubated with 50 μmol/L xanthine, 5 U/mL xanthine oxidase and 1 μg/mL oxidized cytochrome c. The rate of cytochrome c reduction, which is inhibited by the presence of SOD, was monitored for 5 min by reading the absorbance at 550 nm with a Lambda 3 spectrophotometer (PerkinElmer). The results are expressed as μmoles reduced cytochrome c/min/mg cytosolic protein.

### 4.10. Statistical Analysis

Statistical analyses were performed using the two-tailed Student’s *t*-test. All the analyses with a confidence level of 95% are indicated as significant and are marked as follows: * *p* ≤ 0.05; ** *p* ≤ 0.01; *** *p* ≤ 0.001.

## 5. Conclusions

**Figure 4:**
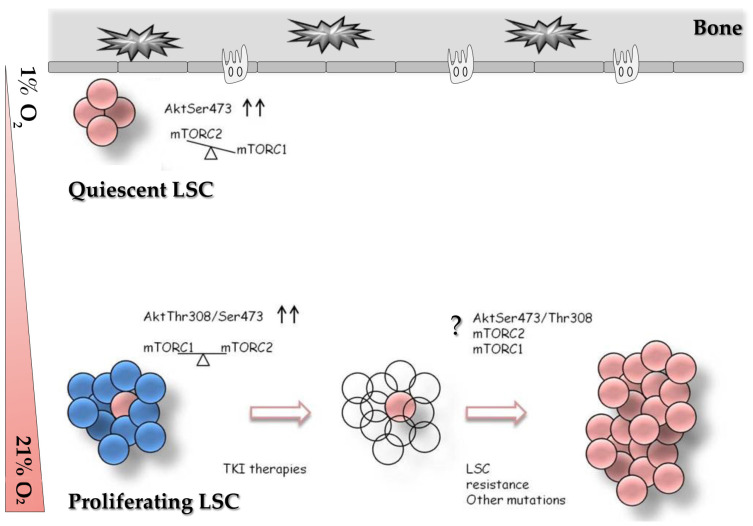
Proposed model of mTORC2 involvement in regulation of quiescent/proliferating LSC. Under low oxygen concentration (1% O2) mTORC2 and Akt (Ser473) could be in an active state (arrows) at the expense of mTORC1. Otherwise, under normal oxygen concentration (21% O2) a balance between mTORC1 and mTORC2, and consequently between Akt (Ser473) and Akt (Thr308) could be detected. The contribute of mTOR complexes on leukemia relapse is unknown (question mark), we proposed the possibility that mTORC2 can have a role in modulate the relapse, in association with other factors, e.g., mutations.

In conclusion, based on our interesting results, we propose a model, as shown in Figure 4, in which the mTORC2 complex is active under hypoxic conditions, in contrast to mTORC1. This imbalance could contribute to the maintenance of the stemness of leukemia cells. In the presence of normal oxygen levels and other signals, LSCs may produce mature blood cells and activate a process of proliferation or the expansion of many leukemic cells. In this regard, a small proportion of LSCs can survive canonical treatment and could play a critical role in CML relapse. In this regard, innovative RNAi nanoparticle-mediated Rictor knockdown has demonstrated specific mTORC2 inhibition in a preclinical breast cancer model, with a successful reduction in crucial cancer features [81]. The identification of the novel pathways involved in the survival of LSCs, such as mTORC2 and its substrates, may provide a therapeutic candidate to exploit in future clinical protocols.

## Data Availability

The following publicly archived datasets were analyzed: https://www.ncbi.nlm.nih.gov/geo/query/acc.cgi?acc=GSE48294 (accessed on 5 December 2019); https://www.ncbi.nlm.nih.gov/geo/query/acc.cgi?acc=GSE43754 (accessed on 5 December 2019).

## Supplementary Materials

The following supporting information can be downloaded at https://www.mdpi.com/article/10.3390/ijms24021234/s1.
