# Supplementary material for: mTORC2 Is Activated under Hypoxia and Could Support Chronic Myeloid Leukemia Stem Cells
Source: Int J Mol Sci. 2023 Jan 8;24(2):1234. doi: 10.3390/ijms24021234 (PMC9865638; doi:10.3390/ijms24021234)
Supplement: Supplementary file 1 [file ijms-24-01234-s001.zip › ijms-2044523-supplementary.pdf]

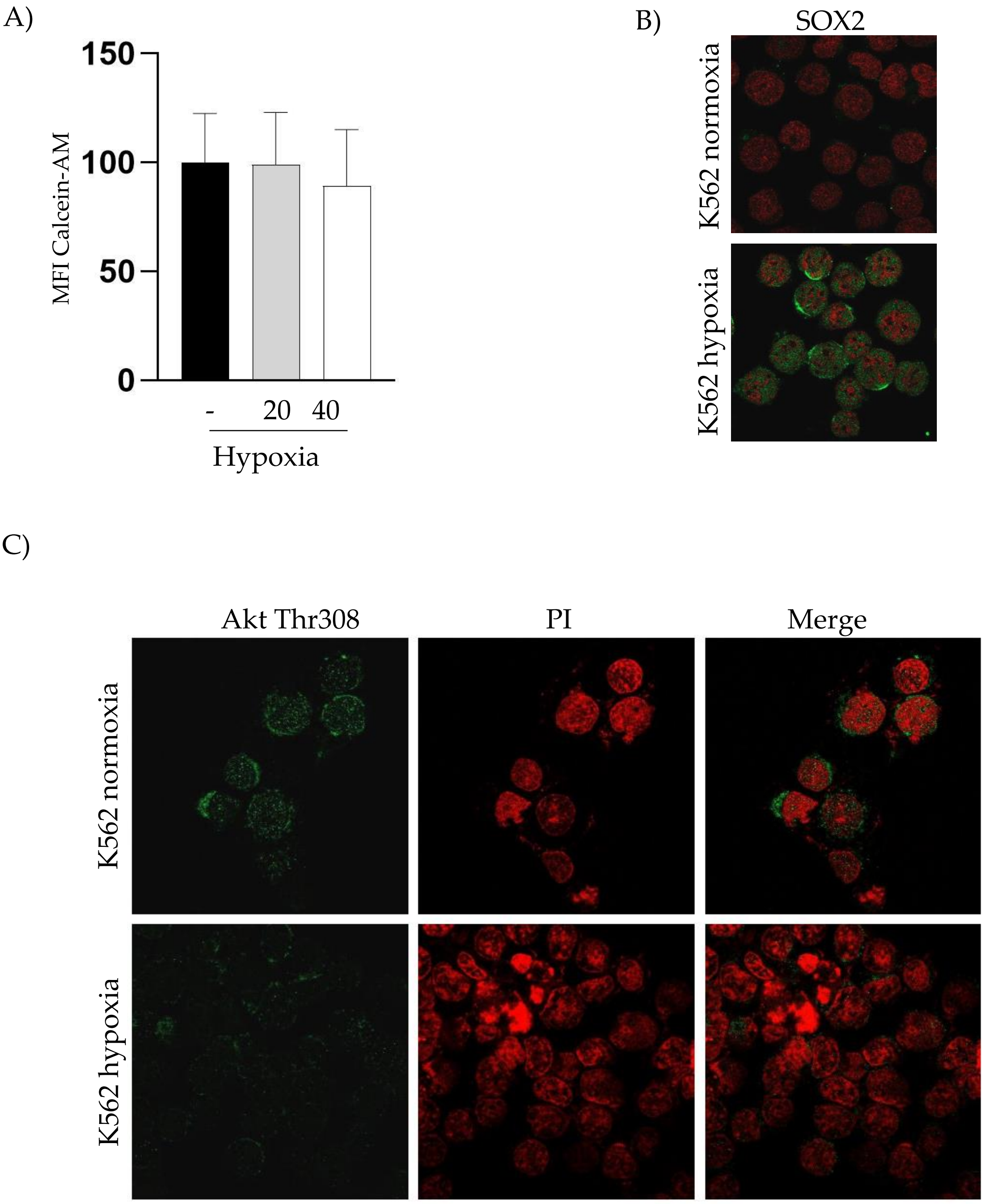

Figure S1: (A) Calcein-AM assay was performed on K562 cells under hypoxic conditions to evaluate the cells viability. The Mean Intensity Fluorescence (MFI) of untreated cells (normoxia) was considered 100% while other conditions were illustrated as % of MFI compared to normoxia (B) Immunofluorescence of Sox2 on K562 cells incubated under hypoxic and normoxic condition. A significant green nuclear staining corresponding toSOx2 was observed after low 1% oxygen incubation, confirming the activation of the stem-cell marker. (C) Immunofluorescence of phospho-Akt (Thr308) on K562 cells incubated under hypoxia and normoxia. A significant reduction of green nuclear staining corresponding to phospho-Akt (Thr308), was observed after low 1% oxygen incubation.

A)

	A	B	C	D	E	F	G	H	I	J	K	L	M
1	NAME	GS<br>follow link to MSigDB	GS DETAILS	SIZE	ES	NES	NOM p-val	FDR q-val	FWER p-val	RANK AT M/	LEADING EDGE		
2	HALLMARK_HYPOXIA	HALLMARK_HYPOXIA	Details ...	127	0.6112539	2.6705027	0	0	0	1042	tags=39%, list=9%, signal=43%		
3	HALLMARK_EPITHELIAL_MESENCHYMAL_T	HALLMARK_EPITHELIAL_MESENCHYMAL_TR	Details ...	85	0.4339258	1.7544385	0	0.0091911	0.026	787	tags=27%, list=7%, signal=29%		
4	HALLMARK_GLYCOLYSIS	HALLMARK_GLYCOLYSIS	Details ...	140	0.3890598	1.6970136	0.0037313	0.0123882	0.054	683	tags=17%, list=6%, signal=18%		
5	HALLMARK_INTERFERON_ALPHA_RESPONS	HALLMARK_INTERFERON_ALPHA_RESPONSE	Details ...	84	0.4095187	1.6516961	0.0071429	0.0160672	0.09	2511	tags=43%, list=22%, signal=55%		
6	HALLMARK_INTERFERON_GAMMA_RESPO	HALLMARK_INTERFERON_GAMMA_RESPONS	Details ...	154	0.3572801	1.596148	0	0.023526	0.165	2882	tags=42%, list=25%, signal=55%		
7	HALLMARK_ESTROGEN_RESPONSE_EARLY	HALLMARK_ESTROGEN_RESPONSE_EARLY	Details ...	111	0.3735961	1.5775162	0.0054054	0.0234877	0.189	2162	tags=30%, list=19%, signal=36%		
8	HALLMARK_HEME_METABOLISM	HALLMARK_HEME_METABOLISM	Details ...	156	0.303265	1.3500512	0.040747	0.1403962	0.777	439	tags=10%, list=4%, signal=10%		
9	HALLMARK_ALLOGRAFT_REJECTION	HALLMARK_ALLOGRAFT_REJECTION	Details ...	113	0.307855	1.3234917	0.0649351	0.1523572	0.837	1968	tags=27%, list=17%, signal=32%		
10	HALLMARK_APICAL_SURFACE	HALLMARK_APICAL_SURFACE	Details ...	18	0.4346905	1.280595	0.1763527	0.1842087	0.904	760	tags=22%, list=7%, signal=24%		
11	HALLMARK_IL2_STAT5_SIGNALING	HALLMARK_IL2_STAT5_SIGNALING	Details ...	140	0.2674887	1.1813724	0.1450512	0.3160103	0.989	787	tags=14%, list=7%, signal=15%		
12	HALLMARK_HEDGEHOG_SIGNALING	HALLMARK_HEDGEHOG_SIGNALING	Details ...	15	0.3966913	1.116006	0.3188119	0.4209367	1	257	tags=13%, list=2%, signal=14%		
13	HALLMARK_KRAS_SIGNALING_DN	HALLMARK_KRAS_SIGNALING_DN	Details ...	56	0.290823	1.0930413	0.2903811	0.4370992	1	1556	tags=23%, list=14%, signal=27%		
14	HALLMARK_XENOBIOTIC_METABOLISM	HALLMARK_XENOBIOTIC_METABOLISM	Details ...	115	0.2467032	1.0440893	0.3898917	0.5145462	1	1059	tags=12%, list=9%, signal=13%		
15	HALLMARK_IL6_JAK_STAT3_SIGNALING	HALLMARK_IL6_JAK_STAT3_SIGNALING	Details ...	53	0.2754277	1.0216026	0.4343808	0.5282354	1	2208	tags=28%, list=20%, signal=35%		
16	HALLMARK_TNFA_SIGNALING_VIA_NFKB	HALLMARK_TNFA_SIGNALING_VIA_NFKB	Details ...	127	0.2254094	0.9632496	0.5239853	0.6352312	1	2265	tags=31%, list=20%, signal=38%		
17	HALLMARK_APICAL_JUNCTION	HALLMARK_APICAL_JUNCTION	Details ...	110	0.1959508	0.8267543	0.8214286	0.9107038	1	2485	tags=25%, list=22%, signal=32%		
18	HALLMARK_ADIPOGENESIS	HALLMARK_ADIPOGENESIS	Details ...	158	0.1686742	0.7565666	0.9643494	0.9666244	1	1569	tags=13%, list=14%, signal=15%		
19	HALLMARK_TGF_BETA_SIGNALING	HALLMARK_TGF_BETA_SIGNALING	Details ...	35	0.1965663	0.6767173	0.9229358	0.9716275	1	1403	tags=14%, list=12%, signal=16%		
20													
21	NAME	GS<br>follow link to MSigDB	GS DETAILS	SIZE	ES	NES	NOM p-val	FDR q-val	FWER p-val	RANK AT M/	LEADING EDGE		
22	HALLMARK_E2F_TARGETS	HALLMARK_E2F_TARGETS	Details ...	180	-0.70922	-3.368198	0	0	0	2000	tags=72%, list=18%, signal=86%		
23	HALLMARK_G2M_CHECKPOINT	HALLMARK_G2M_CHECKPOINT	Details ...	173	-0.652586	-3.071084	0	0	0	2050	tags=60%, list=18%, signal=72%		
24	HALLMARK_MITOTIC_SPINDLE	HALLMARK_MITOTIC_SPINDLE	Details ...	162	-0.491338	-2.287917	0	0	0	2711	tags=46%, list=24%, signal=60%		
25	HALLMARK_MYC_TARGETS_V1	HALLMARK_MYC_TARGETS_V1	Details ...	190	-0.417921	-1.985506	0	0	0	2253	tags=36%, list=20%, signal=44%		
26	HALLMARK_MYC_TARGETS_V2	HALLMARK_MYC_TARGETS_V2	Details ...	54	-0.449951	-1.752858	0	0.0070254	0.026	3879	tags=69%, list=34%, signal=104%		
27	HALLMARK_DNA_REPAIR	HALLMARK_DNA_REPAIR	Details ...	133	-0.385313	-1.751481	0	0.0060628	0.027	2101	tags=30%, list=19%, signal=36%		
28	HALLMARK_ANDROGEN_RESPONSE	HALLMARK_ANDROGEN_RESPONSE	Details ...	78	-0.389411	-1.601433	0.002193	0.024904	0.118	2862	tags=41%, list=25%, signal=55%		
29	HALLMARK_UV_RESPONSE_UP	HALLMARK_UV_RESPONSE_UP	Details ...	106	-0.350581	-1.548984	0.0044248	0.0328167	0.172	2137	tags=32%, list=19%, signal=39%		
30	HALLMARK_PI3K_AKT_MTOR_SIGNALING	HALLMARK_PI3K_AKT_MTOR_SIGNALING	Details ...	78	-0.373893	-1.519946	0.0149893	0.040449	0.229	3198	tags=45%, list=28%, signal=62%		
31	HALLMARK_SPERMATOGENESIS	HALLMARK_SPERMATOGENESIS	Details ...	57	-0.361778	-1.440672	0.0311111	0.0736212	0.402	1539	tags=30%, list=14%, signal=34%		
32	HALLMARK_APOPTOSIS	HALLMARK_APOPTOSIS	Details ...	118	-0.316853	-1.4277	0.020316	0.0745494	0.433	1820	tags=28%, list=16%, signal=33%		
33	HALLMARK_MTORC1_SIGNALING	HALLMARK_MTORC1_SIGNALING	Details ...	176	-0.296294	-1.405342	0.0066519	0.0805248	0.491	3176	tags=48%, list=28%, signal=66%		
34	HALLMARK_OXIDATIVE_PHOSPHORYLATIC	HALLMARK_OXIDATIVE_PHOSPHORYLATION	Details ...	188	-0.288171	-1.382647	0.0072993	0.0889308	0.563	3488	tags=38%, list=31%, signal=54%		
35	HALLMARK_COMPLEMENT	HALLMARK_COMPLEMENT	Details ...	113	-0.303736	-1.339515	0.038961	0.1143607	0.679	1717	tags=23%, list=15%, signal=27%		
36	HALLMARK_REACTIVE_OXYGEN_SPECIES_	HALLMARK_REACTIVE_OXYGEN_SPECIES_PA	Details ...	45	-0.322799	-1.195947	0.1844444	0.2941908	0.967	3360	tags=47%, list=30%, signal=66%		

Figure S2: A) Complete list of gene sets significantly enriched ($p<0.05$) with a positive enrichment score (top of the ranked list) or a negative enrichment score (bottom of the ranked list).
